# Supplementary material for: A scoping review on technology applications in agricultural extension
Source: PLoS One. 2023 Nov 6;18(11):e0292877. doi: 10.1371/journal.pone.0292877 (PMC10627468; doi:10.1371/journal.pone.0292877)
Supplement: S2 File — (DOCX) [file pone.0292877.s002.docx]

Appendix A – Database Search Strategies

# CAB Abstracts (Ovid) Search Strategy, limited to 1/1/2000-10/28/2022

1 educational technology/

2 digital technology/

3 information technology/

4 exp computer software/

5 internet/

6 exp multimedia instruction/

7 audiovisual aids/

8 computers/

9 exp videos/

10 exp distance teaching/

11 social media/

12 ((educat* or instruct*) adj1 tech*).ab,ti.

13 ((webbased or "web based" or web-based or online or on-line or cyber) adj1 (instruct* or educat* or train* or course* or module*)).ab,ti.

14 "mobile app*".ab,ti.

15 (computer* adj2 (educat* or instruct*)).ab,ti.

16 "Intelligent Tutor*".ab,ti.

17 "etutor*".ab,ti.

18 ((intelligent or computer* or computer assisted or artificial or webbased or web-based or online) adj1 tutor*).ab,ti.

19 "video*".ab,ti.

20 (internet adj1 (agricultur* or extension or educat* or teach*)).ab,ti.

21 (computer adj1 (agricultur* or extension or educat* or teach*)).ab,ti.

22 tablet.ab,ti.

23 ipad.ab,ti.

24 "elearn*".ab,ti.

25 "electronic tech*".ab,ti.

26 "distance learn*".ab,ti.

27 "distance teach*".ab,ti.

28 "distance educat*".ab,ti.

29 "wiki*".ab,ti.

30 virtual reality.ab,ti.

31 (VR adj2 tech*).ab,ti.

32 "mobile tech*".ab,ti.

33 flipped classroom.ab,ti.

34 "tech* use".ab,ti.

35 "tech* usage".ab,ti.

36 "digital game*".ab,ti.

37 "game based learn*".ab,ti.

38 "blog*".ab,ti.

39 social media.ab,ti.

40 twitter.ab,ti.

41 facebook.ab,ti.

42 instagram.ab,ti.

43 snapchat.ab,ti.

44 tiktok.ab,ti.

45 whatsapp.ab,ti.

46 "smartphone*".ab,ti.

47 iphone.ab,ti.

48 "mobile learn*".ab,ti.

49 ed-tech.ab,ti.

50 virtual class*.ab,ti.

51 podcast*.ab,ti.

52 microcomputer*.ab,ti.

53 or/1-52

54 exp extension/

55 youth programmes/

56 extension education/

57 "agricultur* extension".ab,ti.

58 future farmers of america.ab,ti.

59 FFA.ab,ti.

60 4-H.ab,ti.

61 4H.ab,ti.

62 (extension adj1 (educat* or teach* or learn*)).ab,ti.

63 "farm* educat*".ab,ti.

64 "youth program*".ab,ti.

65 community development.ab,ti.

66 youth development.ab,ti.

67 (Extension adj1 (agent* or specialist* or professional* or officer*)).ab,ti.

68 (Extension adj1 (program* or service* or system*)).ab,ti.

69 or/54-68

70 53 and 69

71 limit 70 to yr="2000 -Current"

# AGRICOLA (EBSCO) Search Strategy, limited to 2000 to 11/1/22

( ( ZU "educational technology" or ZU "digital technology" or ZU "information technology" or ZU "computer software" or ZU "internet" or ZU "multimedia instruction" or ZU "audiovisual aids" or ZU "computers" or ZU "videos" or ZU "distance education" ) ) OR TI ( ( (education* or instruct*) N1 tech* ) OR (webbased or web based or web-based or online or on-line or cyber) N1 (instruct* or educat* OR train* OR course* OR module*) ) OR “Intelligent tutor*” OR etutor* OR ( (intelligent or computer* or computer assisted or artificial or webbased or web-based or online) N1 tutor* ) OR video* OR (Internet N1 (agricultur* OR extension OR educat* OR teach*)) OR TI ( Computer* N1 (agricultur* OR extension OR educat* OR teach*)) OR Tablet* OR Ipad OR elearn* OR "electronic tech*" OR "distance learn*" OR "distance teach*" OR "distance educat*" OR wiki* OR “virtual reality” OR (VR N2 tech*) OR "mobile tech*" OR “mobile app*” OR flipped classroom* OR "tech* use" OR "tech* usage" OR "digital game*" OR "game based learn*" OR blog* OR “social media” OR twitter OR facebook OR instagram OR snapchat OR tiktok OR whatsapp OR smartphone* OR iphone OR "mobile learn*" OR ed-tech OR virtual class* OR podcast* OR microcomputer* ) ) OR AB ( ( (education* or instruct*) N1 tech* ) OR (webbased or web based or web-based or online or on-line or cyber) N1 (instruct* or educat* OR train* OR course* OR module*) ) OR “Intelligent tutor*” OR etutor* OR ( (intelligent or computer* or computer assisted or artificial or webbased or web-based or online) N1 tutor* ) OR video* OR (Internet N1 (agricultur* OR extension OR educat* OR teach*)) OR TI ( Computer* N1 (agricultur* OR extension OR educat* OR teach*)) OR Tablet* OR Ipad OR elearn* OR "electronic tech*" OR "distance learn*" OR "distance teach*" OR "distance educat*" OR wiki* OR “virtual reality” OR (VR N2 tech*) OR "mobile tech*" OR “mobile app*” OR flipped classroom* OR "tech* use" OR "tech* usage" OR "digital game*" OR "game based learn*" OR blog* OR “social media” OR twitter OR facebook OR instagram OR snapchat OR tiktok OR whatsapp OR smartphone* OR iphone OR "mobile learn*" OR ed-tech OR virtual class* OR podcast* OR microcomputer* ) )

AND

( ZU "agricultural extension" or ZU "future farmers of america" or ZU "4-h" or ZU "4-h clubs" or ZU "agricultural development" or ZU "extension education" or ZU "farmer education" or ZU "youth programs" or ZU "community development" or ZU "youth development" ) OR TI ( “agricultur* extension” OR “future farmers of america” OR FFA OR 4-H OR 4H OR extension N1 educat* OR extension N1 teach* OR extension N1 learn* OR “farm* educat*” OR “youth program*” OR “community development” OR “youth development” OR ( Extension N1 (agent* OR specialist* OR professional* OR officer*) ) OR ( Extension N1 (program* OR service* OR system*)) ) OR AB ( “agricultur* extension” OR “future farmers of america” OR FFA OR 4-H OR 4H OR extension N1 educat* OR extension N1 teach* OR extension N1 learn* OR “farm* educat*” OR “youth program*” OR “community development” OR “youth development” OR ( Extension N1 (agent* OR specialist* OR professional* OR officer*) ) OR ( Extension N1 (program* OR service* OR system*)) )

# ERIC (EBSCO) Search Strategy, limited to 2000-11/1/22

( DE "Educational Technology" OR DE "Instructional Systems" OR DE "Performance Technology" OR DE "Information Technology" OR DE "Web 2.0 Technologies" OR DE "Computer Software" OR DE "Courseware" OR DE "Database Management Systems" OR DE "Search Engines" OR DE "Web Browsers" OR DE "Internet" OR DE "Multimedia Instruction" OR DE "Audiovisual Aids" OR DE "Instructional Films" OR DE "Computers" OR DE "Laptop Computers" OR DE "Handheld Devices" OR DE "Electronic Learning" OR DE "Distance Education" OR DE "Virtual Classrooms" OR DE "Computer Uses in Education" OR DE "Social Media" OR DE "Game Based Learning" OR DE "Video Technology" ) OR TI ( ( ( (education* or instruct*) N1 tech* ) OR (webbased or web based or web-based or online or on-line or cyber) N1 (instruct* or educat* OR train* OR course* OR module*) ) OR “Intelligent tutor*” OR etutor* OR ( (intelligent or computer* or computer assisted or artificial or webbased or web-based or online) N1 tutor* ) OR video* OR (Internet N1 (agricultur* OR extension OR educat* OR teach*)) OR TI ( Computer* N1 (agricultur* OR extension OR educat* OR teach*)) OR Tablet* OR Ipad OR elearn* OR "electronic tech*" OR "distance learn*" OR "distance teach*" OR "distance educat*" OR wiki* OR “virtual reality” OR (VR N2 tech*) OR "mobile tech*" OR “mobile app*” OR flipped classroom* OR "tech* use" OR "tech* usage" OR "digital game*" OR "game based learn*" OR blog* OR “social media” OR twitter OR facebook OR instagram OR snapchat OR tiktok OR whatsapp OR smartphone* OR iphone OR "mobile learn*" OR ed-tech OR virtual class* OR podcast* OR microcomputer* ) ) ) OR AB ( ( ( (education* or instruct*) N1 tech* ) OR (webbased or web based or web-based or online or on-line or cyber) N1 (instruct* or educat* OR train* OR course* OR module*) ) OR “Intelligent tutor*” OR etutor* OR ( (intelligent or computer* or computer assisted or artificial or webbased or web-based or online) N1 tutor* ) OR video* OR (Internet N1 (agricultur* OR extension OR educat* OR teach*)) OR TI ( Computer* N1 (agricultur* OR extension OR educat* OR teach*)) OR Tablet* OR Ipad OR elearn* OR "electronic tech*" OR "distance learn*" OR "distance teach*" OR "distance educat*" OR wiki* OR “virtual reality” OR (VR N2 tech*) OR "mobile tech*" OR “mobile app*” OR flipped classroom* OR "tech* use" OR "tech* usage" OR "digital game*" OR "game based learn*" OR blog* OR “social media” OR twitter OR facebook OR instagram OR snapchat OR tiktok OR whatsapp OR smartphone* OR iphone OR "mobile learn*" OR ed-tech OR virtual class* OR podcast* OR microcomputer* ) ) )

AND

( DE "Rural Extension" OR DE "Young Farmer Education" OR DE "Extension Agents" ) OR TI ( ( “agricultur* extension” OR “future farmers of america” OR FFA OR 4-H OR 4H OR extension N1 educat* OR extension N1 teach* OR extension N1 learn* OR farm* N1 educat* OR ( Extension N1 (agent* OR specialist* OR professional* OR officer*) ) OR ( Extension N1 (program* OR service* OR system*)) ) OR AB ( ( “agricultur* extension” OR “future farmers of america” OR FFA OR 4-H OR 4H OR extension N1 educat* OR extension N1 teach* OR extension N1 learn* OR farm* N1 educat* OR ( Extension N1 (agent* OR specialist* OR professional* OR officer*) ) OR ( Extension N1 (program* OR service* OR system*)) )

# Education Source (EBSCO) Search Strategy, limited to 2000 to 11/1/22

( ( DE "Educational technology" OR DE "Audiovisual education" OR DE "Calculators in education" OR DE "Computers in education" OR DE "Digital badges in education" OR DE "Drone aircraft in education" OR DE "Educational technology industries" OR DE "Internet in education" OR DE "Media programs (Education)" OR DE "Mobile learning" OR DE "Multimedia systems in education" OR DE "Programmed instruction" OR DE "Teaching machines" OR DE "Virtual classrooms" OR DE "Virtual reality in education" OR DE "Information technology" OR DE "Internet in education" OR DE "Computer software" OR DE "Application software" OR DE "Artificial intelligence" OR DE "Children's software" OR DE "Computer assisted instruction authoring software" OR DE "Computer software development" OR DE "Courseware" OR DE "Interactive multimedia" OR DE "Reading software" OR DE "Video games" OR DE "Multimedia systems in education" OR DE "Digital learning" OR DE "Digital learning" OR DE "Computers in education" OR DE "Electronic textbooks" OR DE "Internet in education" OR DE "Mobile learning" OR DE "Online education" OR DE "Social media in education" OR DE "Virtual reality in education" OR DE "Gamification" OR DE "Video games in education" OR DE "Distance education" OR DE "Copyright & distance education" OR DE "Correspondence schools & courses" OR DE "Libraries & distance education" OR DE "Telephone in education" OR DE "Television in education" ) ) OR TI ( ( ( ( (education* or instruct*) N1 tech* ) OR (webbased or web based or web-based or online or on-line or cyber) N1 (instruct* or educat* OR train* OR course* OR module*) ) OR “Intelligent tutor*” OR etutor* OR ( (intelligent or computer* or computer assisted or artificial or webbased or web-based or online) N1 tutor* ) OR video* OR (Internet N1 (agricultur* OR extension OR educat* OR teach*)) OR TI ( Computer* N1 (agricultur* OR extension OR educat* OR teach*)) OR Tablet* OR Ipad OR elearn* OR "electronic tech*" OR "distance learn*" OR "distance teach*" OR "distance educat*" OR wiki* OR “virtual reality” OR (VR N2 tech*) OR "mobile tech*" OR “mobile app*” OR flipped classroom* OR "tech* use" OR "tech* usage" OR "digital game*" OR "game based learn*" OR blog* OR “social media” OR twitter OR facebook OR instagram OR snapchat OR tiktok OR whatsapp OR smartphone* OR iphone OR "mobile learn*" OR ed-tech OR virtual class* OR podcast* OR microcomputer* ) ) ) ) OR AB ( ( ( ( (education* or instruct*) N1 tech* ) OR (webbased or web based or web-based or online or on-line or cyber) N1 (instruct* or educat* OR train* OR course* OR module*) ) OR “Intelligent tutor*” OR etutor* OR ( (intelligent or computer* or computer assisted or artificial or webbased or web-based or online) N1 tutor* ) OR video* OR (Internet N1 (agricultur* OR extension OR educat* OR teach*)) OR TI ( Computer* N1 (agricultur* OR extension OR educat* OR teach*)) OR Tablet* OR Ipad OR elearn* OR "electronic tech*" OR "distance learn*" OR "distance teach*" OR "distance educat*" OR wiki* OR “virtual reality” OR (VR N2 tech*) OR "mobile tech*" OR “mobile app*” OR flipped classroom* OR "tech* use" OR "tech* usage" OR "digital game*" OR "game based learn*" OR blog* OR “social media” OR twitter OR facebook OR instagram OR snapchat OR tiktok OR whatsapp OR smartphone* OR iphone OR "mobile learn*" OR ed-tech OR virtual class* OR podcast* OR microcomputer* ) ) ) )

AND

( DE "Agricultural extension work" OR DE "Agricultural extension worker education" OR DE "Education of agricultural laborers" OR DE "Extension workers" ) OR TI ( ( “agricultur* extension” OR “future farmer*” OR FFA OR 4-H OR 4H OR extension N1 educat* OR extension N1 teach* OR extension N1 learn* OR farm* N1 educat* OR ( Extension N1 (agent* OR specialist* OR professional* OR officer*) ) OR ( Extension N1 (program* OR service* OR system*)) ) ) OR AB ( ( “agricultur* extension” OR “future farmer*” OR FFA OR 4-H OR 4H OR extension N1 educat* OR extension N1 teach* OR extension N1 learn* OR farm* N1 educat* OR ( Extension N1 (agent* OR specialist* OR professional* OR officer*) ) OR ( Extension N1 (program* OR service* OR system*)) ) )

# Web of Science Core Collection, limited to 01/01/2000-12/31/2022

((education* or instruct*) NEAR/1 technolog*) OR webbased NEAR/1 instruct* OR web based NEAR/1 instruct* OR online NEAR/1 instruct* OR webbased NEAR/1 educat* OR web based NEAR/1 educat* OR online NEAR/1 educat*OR “Mobile app*” OR (computer* NEAR/1 (educat* or instruct*)) OR “Intelligent tutor*” OR etutor* OR intelligent NEAR/1 tutor* OR computer* NEAR/1 tutor* or computer assisted NEAR/1 tutor* or artificial NEAR/1 tutor* or webbased NEAR/1 tutor*or web-based NEAR/1 tutor* or online NEAR/1 tutor* OR video* OR Tablet* OR Ipad* OR elearn* OR "electronic tech*" OR "distance learn*" OR "distance teach*" OR "distance educat*" OR wiki* OR “virtual reality” OR (VR NEAR/1 tech*) OR "mobile tech*" OR flipped classroom* OR "tech* use" OR "tech* usage" OR "digital game*" OR "game based learn*" OR blog* OR “social media” OR twitter OR facebook OR instagram OR snapchat OR tiktok OR whatsapp OR smartphone* OR iphone OR "mobile learn*" OR technology OR ed-tech OR (Internet NEAR/1 (agricultur* OR extension OR educat* OR teach*)) OR ( Computer* NEAR/1 (agricultur* OR extension OR educat* OR teach*)) (Topic) and “agricultur* extension” OR “future farmer*” OR “state ffa” OR “national ffa” OR "ffa organ*" OR extension NEAR/1 educat* OR “farm* educat*” OR “Extension agent*” OR “Extension specialist*” OR “Extension professional*” OR “extension officer*” (Topic)
