# Supplementary material for: A scoping review on technology applications in agricultural extension
Source: PLoS One. 2023 Nov 6;18(11):e0292877. doi: 10.1371/journal.pone.0292877 (PMC10627468; doi:10.1371/journal.pone.0292877)
Supplement: Attachment — Submitted filename: comments and response table.docx [file pone.0292877.s003.docx]

| **Reviewers’ Comments** | **Response** |
| --- | --- |
| **Reviewer 1** |  |
| This is a very interesting article. The author has delved deep into the subject matter, presenting well-researched insights and thoughtful arguments. The clarity of writing and logical flow make it an engaging read. The article effectively captures the reader's attention from the beginning till the end. The supporting evidence and references add credibility to the claims made. Overall, it's a valuable contribution to the field. I highly recommend accepting it in the current form. Great work! | Dear reviewer, thanks for your comments. |
| **Reviewer 2** |  |
| **Abstract** |  |
| **General Comments** |  |
| The abstract presented offers an in-depth scoping review of technology's role in agricultural extension programs. The approach is comprehensive, and the narrative effectively captures the integration of both agricultural and educational technologies. The use of multiple databases for sourcing articles provides a robust foundation for the findings. The structured presentation of findings is also commendable. However, certain aspects of the abstract would benefit from added clarity or additional information. Specific details and clearer articulation in some areas would enhance the reader's understanding and make the abstract even more impactful. | We appreciate your valuable feedback, and we want to assure you that we carefully reviewed and addressed each comment. |
| **Specific Comments** |  |
| 1. The abstract briefly touches upon the lack of previous reviews on the impact of technology in agricultural extension. It would be beneficial if the authors briefly indicate why this particular focus is of significance. | We have addressed this matter in lines 9-10. |
| 2. Justifying the choice of the five databases or mentioning if these are the most prominent databases in this field, would enhance the credibility of the study. | We have addressed the matter raised in line 10-13. |
| 3. The mention of India and Africa requires more context. It would be useful to know if this observation indicates a trend or if it merely represents the scope of available literature. | We appreciate your valuable contribution. Under section 5.1 where we discussed the substantive features of the study, we were able to give great details concerning the context of the countries and the regions where our included studies emanated from.  Our study identified studies from 17 different countries such as India, Uganda, Benin, USA and so on, out of which 16 of the 54 studies included in our study were all conducted in India. In lines 296-317 we delved into the trends when we noted that “the prevalence of studies conducted in the predominantly developing countries (excluding USA), is indicative of the high number of farm families in these regions compared to extension services” and noted that “technology therefore plays a crucial role in bridging the gap in effectively reaching a large population of farmers in these areas within a short period.” We also corroborated this with the findings of Antwi-Agyei & Stringer (2021), Idowu (2005) and Boonjing (2008).  However, owing to the 300 word limit for the abstract, we are unable to encompass every intricate detail within it. |
| 4. The distinction between the quantitative research method being the most employed and the mixed methods being the most used data collection approach might be confusing. It would be helpful if the authors could provide a brief explanation or example of this distinction. | Thanks for the suggestion. It has been addressed in lines 17-18 where the mixed methods of data collection specifically refer to the studies that used a combination of 2 or more methods in their data collection process. |
| 5. While the most widely used educational technology is mentioned, the abstract could benefit from highlighting a few of the most common agricultural technologies that appeared in the reviewed studies. | This has been addressed in lines 18-20. |
| 6. The statement that the impacts were "mostly mixed" requires further specificity. Providing a brief example or elaborating on what areas showed positive or negative impacts would be beneficial. | While we would certainly prefer to provide more extensive details, the 300-word limit constrains us. Therefore, we have delved into this matter extensively within the Results and Discussion section. |
| 7. It's commendable to acknowledge potential limitations. A brief mention of one or two key limitations would be insightful. | This has been addressed in lines 24-25 |
| 8. The abstract concludes with an emphasis on gaps in the literature. Mentioning one or two primary gaps or areas for future research would provide readers with a clear takeaway | We touched upon this in the text, however, we were constrained by the word limitation to fully include this within the abstract. |
| **Introduction** |  |
| **General Comments** |  |
| The introduction offers a clear context and rationale for the importance of integrating technology into agricultural extension programs. The progression from the significance of technology in enhancing extension programs to the purpose of the scoping review is logical. The emphasis on the potential benefits for policymakers, researchers, and practitioners provides a broad perspective on the review's relevance. However, some areas could benefit from further elaboration, and the structure might be enhanced to offer a more concise and direct presentation of the main points. | We appreciate your valuable feedback, and we want to assure you that we carefully reviewed and addressed each comment. |
| **Specific Comments** |  |
| 1. The introduction starts strongly by emphasizing the importance of agricultural extension programs. However, it could benefit from a brief mention of the historical or traditional methods of agricultural extension for context. | This has been addressed in lines 33-40 as supported by Vanclay & Lawrence (1994). |
| 2. While the importance of technology in agricultural extension is emphasized, it would be beneficial to provide examples or categories of such technologies. This would offer readers a clearer picture of what technological integrations are being discussed. | This has been addressed in lines 43-46 where some examples of the technologies such as ICTs have been listed. |
| 3. The references (1) and (2) are placeholders. In the final manuscript, it would be crucial to ensure that these references are accurately representing the claims made. | We made sure the citations are exactly stating and supporting the claims. |
| 4. The statement about shedding light on the "current state of research" and mapping the field is clear. However, distinguishing between the broader goals of the review and the specific objectives could provide more clarity. | The aim (broader goal) is addressed in lines 49-50 while the specific objectives are addressed in lines 52-62. |
| 5. The mention of policymakers, researchers, and practitioners is appropriate. Still, it might be enhanced by briefly discussing the specific challenges or questions each of these groups faces that the review can address. | This has been addressed in lines 55-62 where we justified how are research will contribute to the three categories of policy makers, researchers/scientists, and extension agents in the field. |
| 6. The final part of the introduction discusses the research's aims to lay a foundation for future studies. While this is a strong ending, it might be enhanced by presenting a more concise summary of the intended contributions and outcomes of the review | This has been addressed in lines 63-70 where we have summarized the intended contributions of the outcomes of this review. |
| **Literature Review** |  |
| **General Comments** |  |
| The literature review offers a comprehensive overview of the integration of technology in agricultural extension programs. The authors have meticulously categorized the research into the historical perspectives of agricultural extension, the role of technology in agriculture, and the intersection of both. The reference to prior studies and the identification of gaps in existing literature lend robustness to the review. However, some areas could benefit from further clarity, and the structure might be enhanced to offer a more concise presentation of the main points. | We appreciate your valuable feedback, and we want to assure you that we carefully reviewed and addressed each comment. |
| **Specific Comments** |  |
| 1. Agricultural Extension Definitions: The various definitions of agricultural extension provided are comprehensive. However, the transition to the definition that the review aligns with could be smoother. Perhaps a brief rationale for choosing Maunder’s definition would be beneficial. | The specific reasons for choosing Maunder’s definition have been stated in lines 79, and 80-82. |
| 2. Technological Integration: The distinction between agricultural technology as a component of production and as an educational tool is clear. Yet, more explicit connections between the tools and their practical impacts would enhance understanding. For instance, how do drones or IoT directly influence agricultural extension? | This distinction was elaborated in lines 95-109 where the two dichotomous distinctions were made. However, as the primary focus of this stage in the study did not center on the specific breakdown, we comprehensively addressed this aspect in the results section. |
| 3. While the section thoroughly identifies gaps in existing research, it could benefit from a more streamlined presentation. The repeated mention of "technology application in agricultural extension" and the emphasis on the review's unique approach can be condensed to avoid redundancy. | Thank you for your valuable feedback. We have refined this section to improve its clarity and conciseness. |
| 4. Citation and Referencing: The placeholders for references are well-placed, providing a strong foundation for the claims made. In the final manuscript, ensuring that these references are comprehensive and up-to-date will be critical. | The cited references align with the quoted texts and the reference lists at the end. |
| 5. Relevance of Previous Studies: The review does well to distinguish itself from the works of Altalb et al. and Aker. However, a brief mention of why these studies are particularly relevant or how they shaped the current review's approach might provide more context. | Altalb et al. and Aker’s views have been carefully delineated and contrasted with our study that our study diverges from Altalb et al. but not solely concentrating information transfer to farmers, but rather encompasses the broader scope of the agricultural extension system. While Aker also stresses the need to adopt better agricultural technology, it did not delve into the examples of direct TA in agricultural extension.  While both of these perspectives provide a solid starting point, they serve as a springboard for further elaboration within our study. We have substantiated these points in lines 125-145. Your thoughtful comment has helped clarify these critical aspects, and we are grateful for your input. |
| 6. The literature review concludes with a forward-looking statement about enhancing productivity and bridging divides. This is effective but could be enhanced with a brief mention of the expected outcomes or implications of the scoping review. | This has been addressed in lines 142-145. |
| **Research Questions** |  |
| **General Comments** |  |
| The research questions section offers a structured breakdown of the areas that the scoping review aims to address. The categorization into substantive features, methodological features, and characteristics of technology application provides a clear roadmap of the study's approach. The questions themselves are well-formulated and adequately detailed, promising a comprehensive exploration of the topic. However, certain areas could benefit from further specificity or clarity to ensure that the subsequent sections of the manuscript align seamlessly with these guiding questions. | We appreciate your valuable feedback, and we want to assure you that we carefully reviewed and addressed each comment. |
| **Specific Comments** |  |
| 1. While the query about publication information is clear, it might be helpful to specify what particular publication information is of interest (e.g., publisher, year, journal name). Additionally, the inclusion of "agricultural field" is relevant, but the term might benefit from elaboration or examples for clarity. | We have added the two more details about the publication information because those are the ones we considered (lines 152). Agricultural field has been defined in Table 1. |
| 2. The question is comprehensive in covering research methods, data collection approaches, and sample size. However, it might be enhanced by adding inquiries about potential research biases, limitations, or challenges identified in the included studies. | While a scoping review provides a very broad scope, the limitations of the study have already been provided in the conclusion section. |
| 3. The distinction between educational technology and agricultural technology is clear and aligns with the literature review. Yet, the question might benefit from an exploration of the integration or interaction of these technologies. For instance, how does the use of educational technology influence the adoption or effectiveness of agricultural technology? | Thank you for your valuable suggestion, and we recognize its significance. The aspect you mentioned could indeed be a subject for a separate study. We are presently in the process of incorporating it into our future research agenda. To be more specific, we plan to delve into the interaction’s dynamics and its influence on adoption and productivity within the extension system. Your input has inspired us to explore this avenue further. And we also included this in the future direction of this study. |
| 4. The query about the "overall effect of technology on agricultural extension" is broad. It would be beneficial to specify if this effect is being measured in terms of productivity, knowledge transfer, farmer satisfaction, or any other specific metrics. | During our coding meeting, we discussed the possibility of exploring this further. However, since this is a scoping review, we have decided to treat it similar to point (3) and conduct a systematic and meta-analysis to delve deeper into these topics. These research questions are important enough to be studied independently, but they are currently beyond the scope of our scoping review. |
| 5. The research questions set a broad scope for the review. Ensuring that this breadth is maintained throughout the manuscript will be crucial, especially in the results and discussion sections. | We ensured this even as may be noticed in our results discussion section. |
| **Research Method** |  |
| **General Comments** |  |
| The research method section is comprehensive, providing detailed insights into the procedures followed in the scoping review. The use of multiple databases, clearly defined inclusion and exclusion criteria, and a structured coding scheme reflects the systematic approach the authors have taken. The use of PRISMA flow and the description of inter-rater reliability further emphasize the rigor with which the study has been conducted. While the overall methodology appears robust, certain areas could benefit from further clarity or elaboration to ensure the methodological choices are entirely transparent and replicable. | We appreciate your valuable feedback, and we want to assure you that we carefully reviewed and addressed each comment. |
| **Specific Comments** |  |
| 1. It is commendable that the authors have provided the date of the search to ensure the recency of the data. | Thanks |
| 2. While the Appendix A contains the full search strategy for CAB Abstracts, the modifications made to fit other databases would be useful for replication. It would be beneficial to briefly describe or provide these modified strategies in an additional appendix. | We modified Appendix A to include the search strategies for the additional four databases. |
| 3. The criteria are well-defined and comprehensive. However, the delineation between what qualifies as an educational technology versus agricultural technology might benefit from additional examples or elaboration. | This has been further clearly differentiated as seen in lines 177-182 providing a more concise and succinct distinction between the two categories. |
| 4. It would be helpful to know why the authors chose the specific date range of January 1, 2000, to November 1, 2022. While technological advancements since 2000 are mentioned, a brief rationale for this specific range could enhance clarity. | We provided a justification in lines 194-198 where we stated that we chose this time period because we wanted to ensure that “covered the latest studies and documented the rapid progressions of technology in agricultural extension since 2000.” We went further to cite two papers that support this assertion (Purnomo, 2019; Annor-Freempong et al., 2006). |
| 5. The coding scheme is extensive and well-structured. However, the categorization of agricultural field/enterprise could benefit from a more exhaustive list or examples, given that only a few are mentioned. | The agricultural fields listed in lines 214-220 are exhaustively categorized in both the text and Table 1. While coding, we discovered papers from various sub-disciplines that often have related content, even if they are standalone research areas. To maintain standard practice, we decided to group these related sub-disciplines in agriculture. We explained this grouping in the aforementioned lines, grouping them into five major categories: Agricultural economics, agricultural engineering, agronomy, animal husbandry, and mixed studies that cross multiple sub-disciplines. |
| 6. Under the section on methodological features, while the grouping of research methods is clear, a brief rationale for these groupings (especially what constitutes mixed methods) would be useful. | We added a rationale for the use of the quantitative and qualitative categories and clarified our definition of mixed methods. |
| 7. The distinction between educational technology and agricultural technology/innovation is clear. However, the list of technologies and their sub-categories might benefit from further examples or references to ensure clarity. | In lines 247-259 we gave examples of these categories which were all discussed in details in the results and discussion session with various examples and references as seen in lines 427-475. |
| 8. The PRISMA flow diagram, while mentioned, is not provided within the section. If feasible, it would be helpful to include this diagram directly within the manuscript or provide a clearer direction to its location. | Our PRISMA flow diagram was included in the Appendix. |
| 9. The inter-rater reliability is commendably high, but a brief discussion on how discrepancies were resolved (other than the first author acting as arbiter) would provide additional transparency. | We have included this in lines 277-286. Belur et al. (2018) noted that as long as the coding is not super complex, the percentage of agreement between independent coders when their coding is compared could be used and this was what was adopted. |
| 10. It might be helpful to provide a brief overview of the descriptive statistical analyses planned or executed to address the research questions. | This has been added in lines 287-291. We used SPSS to run both the descriptive and cross-tabulation and Chi-square analyses of the type of technology (educational and agricultural) and the effect/impact of the intervention. |
| 11. The section could benefit from a brief discussion or reflection on any anticipated or encountered challenges during the research method, especially during data collection or coding. | This was incorporated into the entire paper section and was discussed in the concussion and recommendation sections. |
| **Results and Discussion** |  |
| **General Comments** |  |
| The Results and Discussion section is extensively detailed, covering a wide range of aspects concerning the substantive features, methodological features, and characteristics of educational technology in agricultural extension. The use of figures, tables, and statistical analyses enhances the rigor and depth of the presented findings. The section is well-organized, with clear sub-sections that aid in understanding the progression of results. However, there are areas that could benefit from further elaboration or explanation to ensure clarity and completeness. | We appreciate your valuable feedback, and we want to assure you that we carefully reviewed and addressed each comment. |
| **Specific Comments** |  |
| 1. The graphical representation of publication distribution (Fig 2) and the breakdown of journals, conferences, and policy papers (Table 2) provide a clear overview of the landscape of research in the field. It would be beneficial to include comments or insights on the top journals or platforms publishing in this area. | Thanks for the suggestion. We added the top journals publishing in this area as may be noted in lines 310-314. |
| 2. The distribution by country and region paints a clear picture of where the research is focused. Some insight into the potential reasons behind the lack of research from certain regions, like Europe or Australia/Oceania, beyond what is provided, might enhance the discussion. | This was stated in lines 324-327 where we stated that “the prevalence of studies conducted in the predominantly developing countries (excluding USA), is indicative of the high number of farm families in these regions compared to extension services. Technology therefore plays a crucial role in bridging the gap in effectively reaching a large population of farmers in these areas within a short period” and also in lines 334-338. we stated that “the dearth of research from Australia/Oceania and Europe in our included studies suggests a need for further investigation in these regions. For instance, Australia/Oceania, renowned for its expertise in animal husbandry due to the combination of large land areas, a substantial livestock population but relatively limited investment in infrastructure and human resources (55), presents a particularly interesting area for future researchers to explore.” |
| 3. The breakdown of research methods, data collection approaches, inferential statistics, and sample size units are comprehensive. It would be interesting to see a further discussion on the implications or reasons behind the prevalent use of quantitative methods over qualitative ones. | Thanks for the suggestion. We added the possible explanation for adopting the quantitative method based on the current research trend/objectives: more confirmatory of the effect of educational technology than exploratory and generalization limitation of qualitative method. |
| 4. The discussion on sample size units and the categorization based on the number of participants adds depth to the results. However, a brief discussion on the implications of these findings for future research would enhance this section. | We added the suggestion of employing diverse sample size units due to the unique characteristics of the agriculture field in future research and investigation of the effect of adopting group/community/households as sample size units compared to individuals in the agriculture extension (lines 391-399) |
| 5. The breakdown of different types of educational technologies and agricultural technologies is detailed and clear. It would be helpful to delve deeper into the reasons behind the prevalent use of certain technologies over others. | We elaborated on this through explaining the characteristics of farmers and the regions (developing countries). Due to their limited educational levels and technology literacy and the funding, resources and social-economic related factors for developing countries. Simple technology such as multimedia and video-mediated are suitable for the targeted population in developing countries in extension.  (lines 429-437). |
| 6. The findings on the intervention characteristics of technology are insightful. The relationship between the duration, intensity, and outcomes of interventions could benefit from further exploration. | We acknowledge that this is an important question. As for investigating the relationship of duration, intensity, and outcomes, there are limitations for us to conduct the analysis. First, there are a significant number of studies that did not report information. (Nine studies (16.68%) did not provide information on the duration, 64.81% (n=35) of the studies did not provide information on the intensity). For those who do report intensity information, some reported precise time while others only provided frequency information. Due to different units to measure intensity and lack of reported information. An analysis of the relationship would be challenging in this study. Moreover, it would be more suitable to investigate the relationship of duration, intensity and outcome in a meta-analysis in which duration and intensity are treated as moderators to see if they moderate effect size. We think a meta-analysis would answer this question better. |
| 7. The cross-tabulation analyses and the Chi-square tests add depth to the results, providing a clear understanding of the relationships between variables. However, some additional interpretation of these results in the context of the broader research landscape would be useful. | This has been addressed in lines 531-561. |
| 8. The section might benefit from a summarization of the main findings and their implications for both researchers and practitioners in the field. | We touched upon this a little in the methods section and gave a summary in the conclusion. |
| 9. While the section is quite detailed, it would be beneficial to see more connections or comparisons with other studies or literature in the field, providing a broader context for the presented findings. | We added the connections and comparison with two previous literature reviews in the agriculture field. We identified some similarities/consistent findings and different focus (lines 563-575). |
